# Southwestern Arabia through the eyes of Western orientalists during the first half of the twentieth century

**DOI:** 10.3389/fsoc.2026.1805886

**Published:** 2026-06-26

**Authors:** Iman Alaadeen I Saigh

**Affiliations:** Department of Arabic Language, College of Arts and Sciences, Najran University, Najran, Saudi Arabia

**Keywords:** orientalism, southwestern Arabia, British travel writing, colonial ambivalence, postcolonial theory, Edward Said, Harry St. John Philby, Wilfred Thesiger

## Abstract

**Introduction:**

This paper discusses the works of Western Orientalists on southwestern Arabia in the first half of the twentieth century. It looks into the ways British travellers portrayed the region in the wider colonial and Orientalist contexts.

**Methods:**

The paper uses postcolonial theories of Edward Said, Homi Bhabha, and Mary Louise Pratt in the analysis of the travel works of Kinahan Cornwallis, Harry St. John Philby, and Wilfred Thesiger. To find the political and cultural representations within these texts, a descriptive-analytical and discourse-based approach was taken.

**Findings:**

The findings indicate that the western interest in southwestern Arabia came up in a colonial intelligence set up that was characterized by British imperial ambitions. Cornwallis was rather an intelligence collector, Philby was a geographical traveler and a political diplomat, and Thesiger was a humanistic and nostalgic depiction of Bedouin life. Their works were colonial ambivalent, with the appreciation of local culture existing alongside political and cultural bias. It is also revealed in the analysis that such stories were determined by the significant historical changes, such as the fall of the Ottoman Empire, and the rise of the Saudi state.

**Discussion:**

The paper has concluded that the writings of Orientalist travels on southwestern Arabia are inseparable with the political and colonial contexts. It suggests the necessity to increase the number of critical studies of Orientalist discourse, translating Western accounts of travels into Arabic, and encouraging more focus on indigenous stories and voices.

## Introduction

The nineteenth and early twentieth centuries witnessed a remarkable expansion of Western intellectual interest in the Middle East, and the Arabian Peninsula in particular, as a space of considerable geographic, historical, and strategic significance. This interest coalesced into what came to be known as Orientalism a broad intellectual movement through which Western scholars sought to study the languages, religions, cultures, and societies of the Islamic East.

Al-Jundi (2016) noted that Orientalism is an intellectual trend concerned with the study of Islamic doctrine, Sharia, Sunnah, Islamic history, and other fields of Islamic studies. This is evident in most Orientalist writings that attempted to understand the cognitive structures of Islamic societies. Despite some manifestations of neutrality, these writings tended to frame the Middle East within European mental frameworks, rooted in a Western-centric civilizational vision (Joseph, 2022).

Edward Said developed the basic criticism of this intellectual tradition as his idea of Orientalism was not only an academic field but a discourse in which Western knowledge about the East was created in a manner that strengthened the structures of colonial authority (Al-lawama, 2024). Later scholarship has developed out of this basis. The idea of colonial ambivalence, the feeling of attraction and repulsion experienced by the colonizer toward the colonized developed by Homi Bhabha can provide a more subtle perspective on the contradictions evident in the writings of these travelers, in which the fascination with Bedouin authenticity was met with coexistence with the degradation of the latter to the object of strategic knowledge (Mousa and Sasani, 2023). The concept of the contact zone as suggested by Mary Louise Pratt, where a social space is established where different cultures come into contact under unequal power dynamics, also sheds more light on the character of the experiences these travelers reported: spaces in which the local actors were not passive receivers of the Western gaze but rather negotiators of their own images (Tarkka and Martin, 2023). Scholars in more modern times, however, have questioned whether this structural play can be avoided even in so-called humanistic descriptions of the Orient. Western Orientalism writing about southwestern Arabia in the context of the present paper is not a review of the region, but rather a set of constructed discourse, guided by the political, institutional, and epistemological horizons of its authors (Asl et al., 2025).

The role of Orientalists, non-Easterners, plays a crucial role in the Western perceptions of the East by studying their Eastern sciences, languages, and literature. They were a result of the existential experience of the West and the Arab world, aiming to cognize the Eastern civilization with the help of writings, translations, and studying literature (Radwan, 2022).

The Arabian Peninsula became a center of attention of the world as a place of colonial rivalry, where travelers with various objectives came. The ensuing literature on politics, society, economics, geography, architecture and archeology was a product of the Eurocentric prism through which these orientalist works were created (Al-Shammari, 2023). The British travel writings of Yemen, e.g., differed significantly depending on the objectives and background of travelers and the individuals they met. Ingrams was a government official that had little knowledge of Arabic, who traveled across Yemen as a colonial officer, whereas Freya Stark was a professional writer, who released many travel memoirs that spanned a diverse Arab landscape (Mannaa and Benlarabi, 2021).

Colonial policies were strengthened through scientific missions: Philip Leibniz and his anthropological and geographical research of southwestern Arabia presented the British with practical information about the life of tribes (Stone III, 2018), and the geopolitical work of Cornwallis in Asir gave British leaders information about the dangers of the Ottoman Empire (ALEtaneh et al., 2024). More humanistic views of Bedouin life and their values and spirituality were introduced by writers such as Wilfred Thesiger in the twentieth century, such as in the Arabian Sands (Walker, 2022; Fookes et al., 2019). Equally, Philby wrote The Heart of Arabia that rendered the tribal organization and political dynamics that formed the initial relations between Britain and Saudi (Owtram, 2023).

These travel texts can thus be situated in the wider rubric of Orientalist knowledge production that is not just a mere record of Arabian cultures but rather an active involvement in the making of a Western-legible Orient selective, hierarchical, and highly embedded in imperial desires (Gani and Marshall, 2022). The value of the work is that it interprets these texts as a whole analytical corpus and not as personal narratives and adopts an integrated discourse-based model, which links Saidian criticism with the ambivalence of Bhabha, the contact zones of Pratt and the political situation of the Arabian Peninsula during the period of 1900 to 1950.

One of the close friends of Ibn Saud was Philby and he played a significant role in shaping Saudi Arabia since he ensured that the political situation was stable and also brought American firms to the Saudi market. His work led to the US oil-concession talks in Jeddah. Another political reason why Philby traveled to southern Saudi Arabia was to expand Saudi influence into the areas that were not directly under British control (Krairi, 2017).

This paper will discuss how Orientalism affected the Arabian Peninsula at the beginning of the twentieth century, how the works of three Western travelers, who were guided by scientific and strategic interests and objectives, provide us with good evidence of how the West formed its own knowledge about the area.

## Statement of the problem

The Arabian Peninsula has been of great interest to the Europeans, especially in the modern times, since the Middle Ages. The western powers increased their endeavors in the region motivated by the need to know about it and eventually to control it. Even now, when the literature on the history of the Peninsula is becoming increasingly abundant, it becomes possible to observe the fact that scholars tend to be general and overlook certain subregions (Mustafa, 2017).

One of the most significant avenues through which Europe aired this interest was orientalism. The writings of Orientalists and Western travelers were more of an amalgamated epistemological framework that aimed at examining the Islamic East in religious, social and geographical aspects. Western traveler literature, as Mahmoud (2017) noted, was a real-life account of the daily life in the Arabian Peninsula, including political, military, and administrative life, economic activities, social traditions, and details of the life of people that were not present in the official historical documents.

The Arabian Peninsula has been a place of attraction to European travelers who want to know and shape the Islamic world since the fifteenth century (Nawab et al., 2022). The Peninsula has been a place that has attracted travelers and orientalists since more than 200 years in connection to the ancient civilizations and its religious values (Editorial Board, 2020).

From this perspective, the current study aims to answer the following main question: How did Western orientalists construct representations of the southwestern region of the Arabian Peninsula in their writings during the first half of the twentieth century? More specifically, it addresses:

1. What were the reasons for Orientalists’ interest in southwestern Arabia during this period?
2. What factors delayed Western travelers’ arrival in southwestern Arabia?
3. How did Western Orientalists characterize and represent the region?
4. How did cultural and political biases shape these representations?

## Literature review

The Arabian Peninsula has long been studied through the lens of Orientalism, with scholars examining civilizations, languages, religions, and literature. Dar al-Ifta (Dar-alifta.org, 2025) considers Orientalism to be the study of the Eastern world in its broadest sense, applied by Western scholars to the Islamic East and its languages, civilizations, literature, and religions.

### Theoretical framework: from Said to post-Saadian scholarship

The book Orientalism by Said (1978) is the invaluable point of departure of any critical discussion of Western images of the Arab world. Said believed that the Orientalist discourse is not an objective academic project but a system of knowledge production, which the West has created, interpreted, and eventually controlled its perception of the East a perception that could not be made without the application of colonial power. As Al-lawama (2024) shows, the intervention by Said essentially re-oriented literary and cultural criticism by making it focus on the questions of power, representation and identity, which dominate Western descriptions of non-Western societies. This framework is the starting point of the current research.

Critics of Said, though, have pointed out that his narrative does not give due space to agency of colonized, ambiguity and contradiction in colonial discourse and unequal dynamics of real encounter. These limitations are overcome by three other theoretical contributions, which are directly used in this study.

To begin with, the idea of colonial ambivalence by Homi Bhabha is the simultaneous yearning and denial of the relationship between the colonizer and the colonized, the desire to idealize, the desire to belittle, which results in paradoxical representations. The partial use of colonial values by the colonized, in the context of Bhabha is not a process of assimilation but is instead subversion. When applied to the current corpus, the concept of ambivalence, as proposed by Bhabha, can be used to explain how Cornwallis was able to admire the tribal loyalty of Asiri and at the same time turn it into intelligence information, how Philby was able to create a personal attachment of extreme depth to the Arabia people, and how Thesiger was able to romanticize the Bedouin lifestyle and freeze it in an eternal pre-mod The idea has been put into fruitful use in a variety of colonial textual situations, such as travel writing beyond the Arab world (Shah et al., 2022).

Second, the notion of the contact zone social space in which Mary Louise Pratt discusses the encounter of disparate cultures in highly asymmetrical power conditions provides a means to analyze the texts not as such, but as results of the encounters under which they were produced. Importantly, the narration of contact zones presented by Pratt demands that they are not one-way: the local actors negotiate, resist, and selectively appropriate in these zones, although the effects of their activity are systematically hidden in the ensuing written statements (Tarkka and Martin, 2023). That Cornwallis collected intelligence at the tribal councils at which he was invited and where he held a natural place of honor, at the Najdi court where Philby worked as an advisor and observer, at the desert camps where Thesiger was with the Rawashid and Bayt Kathir tribes: all these are contact zones in the sense introduced by Pratt, and all have yielded a different type of textual representation. The model has been successfully used in colonial African situations (Yding, 2024).

Third, contemporary research on travel writing has shifted to what Asl et al. (2025) refer to as a beyond Orientalism approach in which all Western travelogs are viewed as equally mired in Orientalist discourse, or in which some of the texts confound and partially undermine the hegemony. This query is directly allusive to Thesiger whose Arabian Sands has been read as a prime humanistic version and as a high-end late-Orientalist version. This paper adopts the stand that it is not only the apparent humanism of Thesiger that is not what absolves his writing of the structural forces of Orientalist representation: it makes up a particular mode of that representation in which nostalgia of an imagined authentic Arabia are a particularly alluring kind of Othering.

These three frameworks Saidian discourse analysis, Bhabhan ambivalence, and Prattian contact zones give the words with which the textual evidence in this paper is read. They determine the thematic arrangement of the comparative analysis and the discovery of both political and cultural bias in each of the accounts of the travelers.

The tourist gaze was introduced by Urry (2000), and it holds that tourists form images of destinations according to their specific cultural expectations, colonial frustrations and power dynamics. The gaze is not non-historical, but constructed from classes, genders and empires, giving the landscape and the community a representational form. This has been extended and refined in more recent scholarship, which highlights the inter-involves of the gaze, and how the host community sometimes resists or challenges it, resulting in reciprocal gazes that complicate the unilateral gaze from the west (Samarathunga and Cheng, 2021). Orientalist material can be interpreted as a component of this vision of authenticity on the part of the Bedouin, accompanied by condescension and colonialist oversight, placing these works in the wider scope of the politics of sight in tourism and colonialism in southwestern Arabia.

### The importance of orientalist writings on southwestern Arabia

The western writers, artists and travelers had been deeply connected to the culture and history of the Arabian Peninsula, but there was a tendency to view it through the lens of an Orientalism romanticism that reinforced perceived stereotypes (Siddiqui and Sujata, 2023). Nevertheless, these prejudices notwithstanding, Orientalist travelers had an influential part in the description of the geography, society, and politics of the region relying on classical sources, Arabic poetry, and local relations to provide in-depth historical knowledge (Al-Abd al-Jabbar, 2016; Nawab et al., 2022). The strategic importance of the extensive and heterogeneous landscape of the Peninsula, comprising trade routes, settlements, and regional dynamics, was stressed in their writings (Shamsuddin and Ahmad, 2020; Al-Majnouni, 2020). Although these narratives continue to be crucial sources, researchers call on people to be cautious, because most of them were influenced by Western institutional interests and need to be counteracted with native views (Rahman, 2023).

Most importantly, no one of these accounts, however rich in empirically, can be read as a clear documentary evidence. Comparing them on the thematic axes, the image of tribal societies, the creation of the physical environment, the treatment of gender relations, and the attitude to modernization, a lot of convergences and divergences are observed between the three travelers under analysis. The Results and Discussion section is organized around these thematic axes, and not in a chronological manner of each traveler, which is how the chapter is structured. This is in line with the methodological suggestion that convergences and divergences ought to be brought into an analytically visible position in cross-textual comparison as opposed to silencing them with chronological discursive presentation.

### The relationship of Western competition to orientalist travels

Travel writing came to be a potent amalgamation of narration and factual observation, and British travelers, such as Doughty, Burton, and Palgrave, wrote extensively about the Arabian Peninsula in vivid detail (Alrashed, 2020). However, according to Said, the stories that were created in the first half of the twentieth century tended to strengthen imperial masculinity and marginalize the voices of women. In this colonial prism, such travel writers as Kinahan Cornwallis did not simply describe the tribal and geographic truth, but also promoted the strategic interests of the British (Alshehri, 2023). Exploration was also sustained by the colonial competition between Britain and France, with political missions by different explorers being quite common (Nawab et al., 2022). In the wake of WWI Britain increased its presence by signing protectorate treaties and Orientalist expeditions (Haider and Khadir, 2025). These efforts were part of broader Orientalist trends studying Arabic, collecting manuscripts, and conducting research often driven by covert political aims (Primkulova, 2017).

The period between 1900 to 1950 was marked with tremendous political change in the Arabian Peninsula and each of the three travelers in the study was immersed in the particular stage of this change. The falling of the Ottoman power in the Hejaz and Asir a phenomenon whose aspects of governance have been followed in detail by Kuehn (2021) generated a vacuum in the political sphere, which the British imperial strategy was in dire need to fill. The mission which Cornwallis sent to Asir was before the Great Arab Revolt and was planned with the exact object of surveying the situation which could either facilitate or hinder British intervention. In 1917, when the embryonic Saudi state (under Abd al-Aziz ibn Saud) was solidifying its power, Philby came to Najd, which was recorded by the British-Saudi diplomatic correspondence as reported by Al-Qahtani (2023). Thesiger crossed the Empty Quarter in the late 1940s when the last years of the British imperial presence in South Arabia occurred, at the same time when protectorate schemes in Aden and the Gulf were both being cemented and disintegrating (Sills, 2021; Wearing, 2024). Identifying these unique political moments is crucial in making sense of the representational decisions made by the travelers, their target audiences, and practical applications in which their reports found usage.

### Reasons for orientalists’ interest in southwestern Arabia

The reasons for Orientalists’ interest in southwestern Arabia were numerous. Al-Abd al-Jabbar (2016) identified social, cultural, and religious motivations. Socially, Orientalists studied social and cultural aspects using written sources for historical study. Religiously, there was eagerness to explore places mentioned in classical and religious books. Culturally, the region’s ancient civilizations and rich manuscripts attracted Western researchers.

The Arabian Peninsula is a region characterized by ancient tribalism and Islamic conservatism, bordered by Egypt, Sudan, the African Horn, and the Bab el-Mandeb (Carapico, 2004). The pivotal role of Oriental travelers in shaping images of Arabs is widely recognized, as nineteenth-century British travelers perpetuated earlier narratives and legitimized Orientalist discourse (Alzahrani, 2018).

### The status of orientalist writings on southwestern Arabia

Orientalist interpretations have been validly criticized but nonetheless, this literature is worth using in the study of the Arabian Peninsula. These productions provide accurate records, detailed drawings and perceptive observations particularly of the southwestern Arabia such that it becomes a key source in historical, geographical and anthropological studies (Al-Abd al-Jabbar, 2016).

### Reasons for the delay of travelers in reaching the southwest

The causes of slowing down of travelers to arrive at the southwest of the Arabian Peninsula in the first half of the twentieth century are multiple and diverse, and comprise the geographical, historical, religious, and social ones.

Western explorers wrote about the bleak weather and sand dunes of Najd, and little understanding of the topography of the area procrastinated Orientalist archeological work (AlOboudi, 2015; Al-Abd al-Jabbar, 2016).

#### Historical reasons

Following World War, I, the southwest Arabian Peninsula experienced several years of political instability caused by the intervention of Britain and restructuring of power in the region. The British separated local rulers and turned sheikhdoms into protectorates in order to safeguard its colonial possessions. A 1926 agreement with Ibn Saud sparked border disputes between Saudi Arabia and Yemen, and the rivalry between Britain and Italy aggravated the situation in the region and caused independent travel to be even more challenging (Haider and Khadir, 2025).

These were not some diplomatic complications but the tip of an iceberg of a more fundamental change of structure. The Ottoman loss of Asir, the Hejaz, and Yemen left a conflicted terrain of which the nascent Saudi state, Idrisi rule of Asir, the Zaydi imamate of Yemen, and European colonial powers would compete to exert influence. Britain needed to build a steady buffer zone around Aden and it had to be constantly fed with intelligence information but it also produced the type of political instability that made traveling dangerous. The archival research on the British and Italian intelligence networks in the Arabian Peninsula by Gilchrist (2024) shows that the competition between the two imperial powers resulted in an information order a system of surveillance and counter-surveillance in which order who was allowed in the region, under what circumstances, and with what intentions. Jones (2022) also demonstrates how British intelligence officers in South Arabia in the 1950s had to work in a tribal environment, which they were able to map, but not to control, and how the granularity of tribal knowledge was both an instrument and a limitation. These results shed light on why even in the case of travelers who were able to access southwestern Arabia in the first half of the twentieth century, freedom of movement was never absolute, and why there are so many descriptions of a feeling that they were working at the border of political tolerance.

The unification of Saudi power complicated the historical situation of the time even more. The interactions between Britain and the newly formed Saudi state were based on instrumentalization and not out-of-hand colonial subordination as the documentary study on the 1919 Najd delegation to London by Al-Qahtani demonstrates, where the guests of the Saudi court were able to access territories that were not under British direct control in a peculiar way due to their informal advisory status. This access did not just happen freely and it was a response to certain political calculations on both sides of what Pratt would identify as a structurally asymmetrical contact zone.

#### Religious and social reasons

Any Orientalist who tries to explain the modern Arab world to Western audiences encounters great threats, especially thanks to the ingrained prejudices and shallow understanding of Islam among the educated Westerners (Pircher, 2023). These challenges were enhanced by cultural and religious seclusion of parts such as the Hejaz that strongly opposed foreign invasion.

## The most prominent orientalists in southwestern Arabia

### Philip Leibniz and his colleagues

A major discovery of the expedition led by Philip Leibniz to Central Arabia, was a large set of ancient rock carvings with stylized human and animal figures that represented cultural expressions thousands of years ago. Similar results are found by Andreae et al. (2021), who present the development of rock art in central Saudi Arabia, revealing a series of four major cultural periods of evolution: Early Hunters (Neolithic), Hunting and Pastoral, Literate (Iron Age), and Islamic periods. They were mainly discovered in a mountainous area between Asir and the Empty Quarter (Eldosouky et al., 2022).

This expedition had a number of goals: topographical and archeological recording of regions that had never been explored before; the discovery of ancient inscriptions and cultural icons, a creation of knowledge that could be used to benefit a European geo-cultural agenda. The project, involving pioneers like John Philby, Jack Rickman, and Cuszczak Rickman, aimed to create a knowledge database for European Orientalist discourse aligned with future research and political ambitions (Baumer, 2022).

### British orientalist Kinahan Cornwallis: Asir before World War I

Cornwallis’s account of Asir, compiled around the outbreak of World War I, was described by subsequent scholars as of great historical value to students of the Arabian Peninsula (Alshehri, 2023). Before WWI, travelers in Asir viewed their journeys as practically motivated. Cornwallis’s work portrays travelers as instruments for future exploitation.

A close examination of the account of Cornwallis shows that there are three structurally important patterns of representational. To begin with, the text always subjected social observation to strategic classification: descriptions of sheikhs and tribal rulers are written in terms of evaluations of reliability, loyalty, and possible military utility instead of the interest in a social logic of tribal power as such. Descriptions of how much a chief has influence over his men, or of how navigable a certain route to a valley is, give one a clue to the intelligent man behind the traveling character. Second, it can be said that Cornwallis uses what could be considered a calculative ethnography: his accounts of Asiri peoples are accurate and detailed, but the accuracy is enumerative and not comprehensive. People are mapped, roads are categorized, water bodies are found the landscape and its people turned into dots on a strategic map. Third, and above all, pertinent to the context of Bhabha in his colonial ambivalence, the text of Cornwallis swings between expressions of admiration at the stamp lessness and autonomy of the Asiri tribes and a clinical detachment which treats them as objects to be handled, not as objects to be understood. This swing the concomitant idealization and instrumentalization of the Other is the structural mark of ambivalence of colonial discourse (Shah et al., 2022). Not only the content of the account but its grammar of representation was influenced by the intelligence objectives of the mission.

Cornwallis aimed to create a comprehensive map of key roads, tribe names, and sheikhs, assess the military capabilities of Ottoman forces in the region, and pave the way for a greater British political role. The report, written during Britain’s preparations for the Great Arab Revolt, was considered strategically significant. Cornwallis’s role as planner and liaison officer provided accurate field knowledge enabling Britain to influence events to its advantage.

### Wilfred Gilbert Patrick Thesiger Arabian Sands

Wilfred Thesiger, a renowned British explorer, gained international acclaim for his evocative depictions of the Arabian Desert in Arabian Sands (1959). Born in Addis Ababa in 1910, his early exposure to Abyssinia fueled a lifelong passion for exploration (Banks, 2016). Arabian Sands stands out as a profound ethnographic and geographic travelog, drawn from his 10,000-mile journey by camel through the Empty Quarter among tribes like the Rawashid and Mahashar. The book meticulously documents Bedouin life customs, values, and survival methods just before the onset of modern transformation.

The tension between the ethnographic specificity and the nostalgic idealization of thesifier is worth analyzing as it is the key feature of his prose. His records of Rawashid guides and friends are very personalized he records names, family ties, physical traits, personal records and histories in a manner that is quite different to the more aggregative lists of tribes of Cornwallis or the geographical systems of Philby. This individuation, however, acts in conjunction with a programmatic temporality which places Bedouin society in a fading away past. Episodes like accounts of a journey made before the arrival of the oil age, or cries that the old ways were soon to be forgotten, create the Bedouin world as a prehistoric a world as soon as the reader is tempted to mourn over it instead of encountering it as an equal. In the sense of belatedness used by Behdad (borrowed here through his notion of belatedness) Thesiger comes in always already too late, and the sense of belatedness permeates the representational form of the text. Moreover, the almost complete lack of women in the story of the desert journey told by Thesiger even though women are the heart of Bedouin family and economic life is indicative of the gender aspects of his Orientalist concept. Similar to the example of Alrashed (2020) and Siber (2025), both of whom create the desert as a masculine environment of heroic trials, the presence of women was eliminated or minimized to peripheral commentary, the same way imperial travel writing always created the desert as such a masculine setting. This deletion, is not a coincidence in Thesiger, but a discourse characteristic of the discourse in which he writes.

Unlike traditional Orientalist accounts, Thesiger’s narrative offers an alternative representation grounded in respect and lived experience, warning against the loss of authenticity in a rapidly modernizing world. During his 1946–47 expedition, he collaborated with the city’s governor and traveled with thirty men from the Bayt Kathir tribe. Thesiger explored remote, state-absent regions using camels, emphasizing the hardships of desert travel (Thesiger's Journeys in Arabia, 2025).

### Harry St. John Bridger Philby the heart of Arabia

Harry St. John Philby was a leading British explorer and authority on the Arabian Peninsula. His trip to Najd in 1917, which was described in his book The Heart of Arabia, provided an insight into the early development of the Saudi state and cultural and political dynamics in the region (Al-Rodhan, 2017). His contributions earned him the Founder’s Medal from the Royal Scottish Geographical Society (Rsgs.org, 2023).

The text by Philby is performed by a kind of a very sophisticated practice of double writing where a geographical and anthropological surface masks or, to the contrary, facilitates a political and an intelligence subtext. His account of the Najdi scenery, wadis, oases, and routes, is given with the accuracy of a geographical survey, but the system of organization is always strategic: which routes lead to the interior of Arabia and the Gulf coast, which oases may be of service to the movement of the military, which tribal confederations are on the side of the Ottoman or the British. This two-sidedness of the scientific facade on the political basis is, perhaps, most evident in the manner in which Philby discusses Ibn Saud himself. The attitude that Philby has towards the Saudi monarch is a mixture of real political admiration and a steady line of putting Ibn Saud into perspective as someone whose ambitions to dominate the region can be brought to bear on the British interests. It is the zone of mutual instrumentalization that the Riyadh court in the meaning of Pratt that the court required Ibn Saud to gain access to and legitimacy and Ibn Saud required Philby to gain entry to the British diplomatic networks. The product of this uneven, yet two-way negotiation is evident in the textual product of this relationship The Heart of Arabia. The fact that academic knowledge production was entangled with colonial policy, as Gani and Marshall (2022) claim more generally, is not an incidental phenomenon of Orientalist scholarship but a defining feature thereof; Philby exemplifies the entanglement of this phenomenon in a particularly striking way.

Philby’s book encompasses several objectives: providing a comprehensive geographical description of the Arabian Peninsula through the eyes of a geographer; narrating social, cultural, and tribal observations with unprecedented ethnographic detail; analyzing the political situation within a hidden colonial context; and enhancing Western understanding of the region to serve political interests. Although the book appears scientific/geographical, Philby played a dual role as traveler and political advisor.

As shown in Table 1, though each of the travelers used a dissimilar main means of representation, all three of them continued to be entrenched in different forms in the structural processes of Orientalist discourse. The comparative scheme shows that the distinctions between them are not those between Orientalism and the lack thereof, but between various forms of a common representational order.

**Table 1:** Comparison table.

Traveler	Main objective	Focused region	Type of bias	Theoretical lens applied
Kinahan Cornwallis	Intelligence/Colonial Strategy	Asir (pre-WWI)	Political calculative ethnography; tribal admiration alongside instrumentalization (Bhabha’s ambivalence)	Contact zone: tribal councils as intelligence-gathering sites
Harry St. John Philby	Political–Geographical–Anthropological	Najd & Southwestern Arabia	Political/Cultural geographical surface over strategic subtext; dual role as traveler and advisor	Contact zone: Riyadh court as space of mutual instrumentalization
Wilfred Thesiger	Humanistic–Cultural	Empty Quarter & South	Cultural nostalgic idealization; temporal framing of Bedouin society as disappearing; erasure of women	Late-Orientalist ambivalence: humanistic respect alongside structural Othering

## Previous studies

### Arab studies

Studying German Orientalism and its perception of Arab-Islamic civilization, Kaddru (2023) found that there are several trends in Western Orientalism, with one side being more objective and fairer, and the other being more one-sided and biased. Al-Dhalimi (2019) critically examined historical and cultural matters in southern and western Arabia by the writings of German Orientalist Werner Kaskel who concluded that his Lihyanite inscription analysis had not been properly documented. Trosch (2022) investigated the contributions to defining Arabic geographical literature by Russian Orientalist Krachkovsky and French scholar Andre Miguel.

Comparative study of these Arab works shows that there is a systematic issue of concern as to how to evaluate the discourse of Orientalism, yet on the other hand there is the issue of how to rate individual Orientalists on a fair/bias scale without necessarily addressing the structural conditions that bring about both tendencies. The current work transcends this dichotomy by focusing on such discursive processes as ambivalence, selective representation, the contact zone where bias is created and naturalized in texts that can be marked by both a sense of authentic scholarly or humanistic impulse.

### Foreign studies

Elsheikh and Habiba (2023) explored how Orientalist literature affected Western people about the Islamic religion and Arab people and concluded that the Orientalist concepts had a negative influence with religious, colonial, political, and commercial prejudices. The analysis of Yemeni sources of the early twentieth century by Mannaa and Benlarabi (2021) has shown that the British travelers wrote documents about Yemen, which was not characterized by stereotypical Orientalism but was also a depiction of everyday life, customs, and traditions. Siddiek (2017) examined the role of Western Orientalism in the Islamic world, concluding that it played a significant role in facilitating cultural invasion and promoting political interventions.

The foreign studies sources sampled here are especially useful in recording the broadly based impacts of Orientalist discourse, but are inclined to concentrate on the results of the images created, the social opinion molded as opposed to the textual processes by which these results are created. The contribution of this study is precisely the consideration of those mechanisms, through theoretical tools of Bhabha and Pratt, of how particular textual choices what is described and how, what is omitted and why make up the representations of southwestern Arabia that these previous studies only document at the level of effect.

### Research gap

Critical analysis of past studies shows that despite significant literature on Orientalism and Western travel literature on the Arabian Peninsula, most work is either historical-descriptive or broadly ideological in its critique. Very little has focused specifically on southwestern Arabia as a distinct geographical and socio-political region, and fewer still have applied a unified discourse-oriented framework that integrates Saidian critique, Bhabhan ambivalence, and Prattian contact zones to a comparative corpus of texts from a single historically defined period. The present study addresses this gap.

### Methodology

This study adopts an interpretive philosophy, viewing Western travelogs as discourses imbued with cultural and political biases rather than neutral descriptions. This philosophy enables analysis of the meanings and contexts underlying these texts and their connection to theories of Orientalism and postcolonialism.

The analysis procedure was composed of three steps. The first stage was performed as all three main texts were read thoroughly to have a wide range of knowledge about their content, structure, and rhetorical patterns. This preliminary reading was not informed by any predetermined categories but was geared towards tracing some recurring descriptive patterns, conceptual framings and silences aspects of the texts that are systematically missing or underrepresented.

At the second level, thematic coding was used to analyze the three texts, grouping the repetitive motifs in four thematic axes, including tribal social patterns; environmental and geographical description; gender relations; and modernization attitudes. They were not foisted onto the texts but appeared inductively of the texts themselves, through the recurrence of specific vocabulary, modalities of description, frame-evaluations. The resolution to have the comparative analysis conducted on a thematic basis and not chronologically, traveler by traveler was taken exactly to make convergences and divergences analytically visible as necessitated by a strict comparative methodology.

In the third level, the coded content was put through a differentiation of two types of bias. Political favoritism was detected when a description was used to serve colonial or intelligence purposes to map out roads, evaluate the military potentials, and evaluate tribal allegiances to their strategic exploitability. Cultural bias was found where a representation diminished a community or practice to a stereotypically or romanticized image of the timeless Bedouin, the pre-modern desert, the missing woman. This difference is not limited; political, and cultural prejudices in most cases are interconnected and mutually supporting. The difference, however, offers a methodological foundation to the coding which brings the approach to the textual analysis to the level of provable evidence rather than impressionistic description.

Cornwallis, Philby and Thesiger were chosen by strict criteria: direct interest in the southwestern Arabian Peninsula as outlined in this paper; time within the period of 1900–1950; evidenced political or scholarly impact of the texts that result thereof; and accessibility of primary material to close study. Other travelers who were present in the time and area were also present such as Bertram Thomas, whose work was more of a literary and intellectual, as opposed to a political, interest and Amin al-Rihani whose work was too literary and intellectual to be within the geographical and thematic area of this research. The three chosen travelers can be seen as three analytically different modes of the intelligence-political, geographer-diplomatic, and humanistic-critical modes of Orientalist engagement which are at once diverse enough to be studied comparatively and at the same time united by their common regional object.

The study employs an inductive approach, tracing phenomena within the travelers’ texts and arriving at general conclusions about the representation of southwestern Arabia. It also utilizes a descriptive-analytical approach to describe the characteristics of the texts and critically analyze them to uncover patterns of bias and representation.

Recent research has shown that Western tourism to the Arabian Peninsula in the first half of the twentieth century was an emerging trend that had an evident political and cultural aspect. According to there were over thirty known expeditions that occurred in the period between 1900 and 1950. Nawab et al. (2022) prove that the motivations were distributed in the following way: political and intelligence targets (about 45%), geographical targets (30 percent) and cultural and anthropological targets (25 percent). These statistics demonstrate that the works of the Western travelers were not just the subjective reflection of personal experience but were associated with the greater purpose that was comprised of colonialism and anthropology.

## Results and discussion

### First question: reasons for orientalists’ interest

At the beginning of the twentieth century, European colonialism, most notably between Britain and France, and the geopolitical importance of the region as the entrance to the Arabian Gulf and Indian Ocean, affected interest in southwestern Arabia. Western powers were aware of the strategic value of southern Arabia, and exploration and writing expeditions began, which were both documents on cultural knowledge and intelligence sources.

The major ones were political and colonial objectives. Orientalists were a soft front to Western influence; their writing and memoirs primed the political groundwork to the arrival of Europeans, through reports like those of Cornwallis and Doughty, accurate information of tribal allegiance, resource sites and topography. Alshehri (2023) indicates that these reports played a central role in supporting British plans in the Arabian Peninsula.

Documents have verified that the Orientalists were involved in intelligence operations in the pretext of traveling or conducting scientific studies in collaboration with Ministries of War and Colonial Affairs. The justification of journeys aimed at the collection and important intelligence was based on academic means geography, literature, and anthropology, which Gani and Marshall (2022) define as one of the structural characteristics of the relationship between the production of colonial knowledge and the imperial policy in various situations. The publishing houses in the West promoted the popularity of the Orientalist work due to the fascination of the European people with the enigmatic East, which solidified the stereotypes and myths of Western dominance (Rahman, 2023). Travel writing presented the Arabian Peninsula as the country of male heroism and tribal courage, and downplayed the role of women and reflected colonial ideologies (Alrashed, 2020; Siber, 2025).

### Second question: reasons for delay in reaching the southwest

The lateness in the arrival of Westerners in southwestern Arabia could not be attributed to lack of motivation but to a complicated mix of landscape, political situations and social rejection of the cultural conqueror. The area continued to be difficult to explore because of its symbolic and real autonomy and doubts about the motives of travelers be it scientific, intelligence or religious.

The emergence of later, rich texts in the history of travel in the southern Arabian Peninsula was contingent on changing political circumstances and travelers’ success in gaining local communities’ trust or seeking protection from colonial powers. To comprehend this history, one should pay attention to the overlap of the geography, power, and religion.

### Third question: the perception of southwestern Arabia

#### Tribal social structures

The three travelers extensively interacted with the social organization of the tribes although their framings were revealing in different ways. The treatment of Asiri tribes by Cornwallis was basically taxonomic: sheikhs were designated, their retinue was counted, and their loyalty was evaluated on whether they could be aligned to the British side or not or whether they could become a threat to the Ottoman rule. The social logic of tribal authority, its ancestral bases, its traditional law, its internal struggles, interested only insofar as they were subjects of strategic calculations. The description of Najdi and southern tribes by Philby was truer to ethnography in its detail, and gave us the description of dialect differences, of practices of that tribe and of its relations with other tribes, and something of the dualism of geographer and political commentator. But tribal organization here as well came to be interpreted through the prism of politics: which confederacies were pro-Ibn Saud, which were independent, and which could be leveraged or be countered by the British policy. Thesiger had the most personalized approach to the Rawashid and Bayt Kathir, as the 3 months of traveling together and spending most of their time together provided the basis of this relationship. However, his account of tribal society was prismed through a nostalgic lens which had a tendency to solidify the Bedouin social structures as pre-modern and resistant to historical processes of sedentarization, state-building, oil-production, which were already occurring at the time of his journeys.

#### Environmental and geographical characterization

The physical setting of southwestern Arabia was characterized by all the three travelers, which was influenced by their intentions. Terrain The navigability of the wadis, the defensibility of passes in the mountains, the position of water sources, were all terms in language borrowed by military geography (Cornwallis, the origin of which is owning to his military geography). The geographical descriptions by Philby were more methodical and matched the accuracy of a professional surveyor with an interest in the climate, vegetation and geology, which truly were scientific in nature, and earned him praise at the Royal Geographical Society. Thesiger’s treatment of the desert landscape was the most esthetically elaborate: his descriptions of the Empty Quarter’s sand dunes, light conditions, and physical extremity are rendered with a literary intensity that has been widely admired, but they also serve a representational function constructing the desert as a space of absolute authenticity, outside history and modernity, in which the Bedouin can be encountered in their “true” state.

#### Gender relations

The way that the subject of gender is brought to bear in these three accounts is more than impressive in its consistency, rather than the explicit treatment of this subject. All the three travelers succeeded in eliminating women in their major discourse of the area. The intelligent oriented description of Cornwallis finds no place in which women have a place in the life of the tribes, which is described as a purely masculine world of chiefs, warriors and strategic resources. Philby was also interested in ethnography in the form of dialect, customary practice and material culture but he only mentions women marginally in his narrative in the form of people who he came across in a distant place or those that were mentioned in connection with their male relatives. Thesiger has probably been the most important erased, as he purports to have lived in and really known Bedouin society: the women of the Rawashid and Bayt Kathir communities are virtually missing in a story which claims to be a thorough account of desert life. This systematic erasure, as shown by Alrashed (2020) and Siber (2025) was not an accident, but it was a systematic imperial travel writing that has continually created the desert as a manly space of heroic self-testing, and the absence of women reinforced this creation. The implications to historical knowledge are far-reaching: 100 years of Western historical writing on the Bedouin of southwestern Arabia has been constructed on sources which systematically denied a half of the population objectification.

#### Attitudes toward modernization

The three travelers diverged most clearly in their treatment of modernization and change. Cornwallis’s pre-WWI account predates the transformations that oil discovery would bring and engages with the region as a largely static political landscape. The writings of Philby extend over a greater time span and are more ambivalent: he welcomed the unification of the power of the Saudi state, which he had aided in making possible, but was ambivalent regarding the economic and social upheavals that ensued. Thesiger was the most ideologically aggrieved and his stance the most fundamental: Arabian Sands is built around an essential conflict between the primitivism of Bedouin values of endurance, hospitality and independence which he so admired and the modernization which he felt was inexorably ruining it. This resistance has been most commonly recognized as being typical of later Orientalism: a discourse that laments what it itself hastens, recording the fading away of the traditional world just as it is being altered. Even traveling writing that directly criticizes Western modernity, as Asl et al. (2025) note in a similar situation, can be stuck in the representational systems of Orientalism, as long as it represents its subject as passive actors of historical change instead of active participants in it.

Cornwallis, Philby, and Thesiger illustrate how their depictions of Southwestern Arabia are firmly rooted in the Western imagination. Cornwallis reshaped the tribal system into intelligence maps, indicating surveillance. Philby’s diplomatic and geographical publications also demonstrate a political perspective that was oriented toward an understanding of the relationship between empire and emerging Saudi power.

Thesiger’s romantic image of Bedouin life is a reminder of a romanticizing perspective that captured Arab society in a timeless past. From these examples it is clear that travelogs were not mere reports but acts of “seeing” with a discursive quality that were influenced by colonial and cultural perspectives. These descriptions, reflecting Urry's (2000) notion of the tourist gaze, demonstrate how Western visitors formed images of Arabia by selectively observing what they wished to see, whereas later scholarship places focus on the agency of host communities, in the form of an equally reciprocal gaze (Samarathunga and Cheng, 2021).

### Fourth question: cultural and political biases

A cultural and political prejudice is evident in Western Orientalists such as Harry St. John Philby and Wilfred Thesiger, who visited southwestern Arabia in the early twentieth century, which not only added to their images but also led to the formation of an effective Orientalist discourse. These stereotypes were rooted in the fact that they were linked to Western colonial agendas, as well as the European perception of the East as being Other.

The functional colonial point of view, based on political bias, is most pronounced in Cornwallis, who was instilled in the institutions of British intelligence and imperial geography. Yet it can also be found in Philby, whose reports on the field about the tribal structures, trading routes and sources of wealth were organized and presented to the British government. Their ideas about the area were more political than intellectual that informed the British decision-making in the Gulf, Yemen, and the Hejaz as seen in the reports of Cornwallis about Asir (Gilchrist, 2024; Jones, 2022).

Cultural bias the stereotype of the Eastern Other was most visible in Thesiger. Influenced by European perceptions of the Arab world as backward, religiously conservative, and resistant to modernization, his Arabian Sands highlighted the masculine and heroic aspects of Arab desert life while omitting women’s roles, reflecting a supremacist, masculine perspective rooted in the European colonial context (Alrashed, 2020).

Cognitive Bias Selectivity bias selectivity in Representation was the selectivity that was selected in all three accounts. Orientalists were guided by picking on elements of life which suited their narrative purposes, and ignoring the genuine cognitive or scientific aspects dimensions of local culture. Orientalist interpretations of the Arab cultural production, as Rababah et al. (2025) illustrate in an analogous context of analysis, are prone to filter the topic of their analysis through already established ideological frameworks and choose what confirms and excludes what complicates them. The travel stories were not just the narratives about the actually existing world; but they were aimed at creating a stereotypical image that was to answer the Western need needs to know the East in terms of power and control as Edward exactly what Said identified as the logic of Orientalist discourse. Another dimension is the contact zone model of Pratt which introduces the idea that such biases were not created in a vacuum but rather in the circumstances of the encounter, where local actors, guides, and informants had pre-determined what the traveler was able to see and know, whereas the account of the traveler systematically eliminated their presence in the creation of those biases (Yding, 2024; Tarkka and Martin, 2023).

## Conclusion

The paper drew some important conclusions that explain how Western Orientalists created the south western Arabian Peninsula in the first half of the twentieth century and explain the intellectual, political and cultural aspects that prevail in their works.

On the one hand, Orientalist discourse was not just a geographical or social description but went to the political and cultural vision of the formation of Western perceptions of the inhabitants of the region and the essence of local society. These three travelers Cornwallis, Philby, Thesiger, were intelligence, geographer-diplomat and humanist critic of modernity respectively, representing a variant of this discourse that was determined by the political conditions of their respective times.

Second, using the theoretical frameworks of the colonial ambivalence of Bhabha and contact zone of Pratt to these texts, it can be seen that the internal contradictions of each of the accounts cannot be considered incidental, but rather structural. The contempt and admiration that Cornwallis displays in his tribal portraits, the scientific/political intelligence and the humanistic admiration of the Other that pervades Philby’s geographical histories, and the ambivalence between the humanistic respect and nostalgic Otherness that is inherent in the discourse of Thesiger all represent the ambivalence which is so characteristic of colonial discourse at its most advanced levels.

Third, the comparative analysis of the themes of the three accounts of tribal social organization, environmental characterization, gender relations, and attitudes towards modernization demonstrates both important convergences and differences between the three accounts. The systematic obliteration of women in all three stories is most notable, and suggests an overall aspect to imperial travel writing that surpasses the personal differences of the travelers themselves.

Fourth, the paper shows how the spheres of contact where these travelers carried out their business in Asiri tribal councils, Riyadh court, desert camps of the Empty Quarter were not unidirectional Western projection spaces, but instead spaces where local actors had actual (although unfair) control over what the traveler could reach, see and ultimately write. To recover this local agency, there is a need to read against the grain, i.e., not as much as is said but as much as is conspicuously omitted and as much as would result in both.

Lastly, the findings underscore the critical discourse analysis as a method of studying these historical texts, the concealed ties between text and context, and the intellectual and political content on a level that recuperates the logic of structure of representation. The work paves the way to conduct further studies on the influence of Orientalism on cultural and political images on Western minds related to the Arabian Peninsula and other countries.

In summary, Cornwallis’s, Philby’s, and Thesiger’s writings are not travel reports, but were part of a broader western view which helped to shape the picture of Southwestern Arabia in the early part of the twentieth century. It was a look that was at once jumbled and political, diplomatic, romantic, and oblivious to women and to the exigencies of colonial rank. Recognizing this, an Orientalist text was viewed as an instrument of vision and power, and its images persist in the present.

Furthermore, these Orientalist texts could be analyzed via the tourist’s perception as envisaged by John Urry’s theory of the tourist gaze. The travelers were not just describing what they observed, however, creating images of Southwestern Arabia in which they created a segment that accommodated the western desires of exoticism and surveillance as well as a nostalgia that plays with the image of the natives with such beautiful faces. This field of vision was instrumental in making colonial realities palatable and in perpetuating structures of power while constructing the imaginations of the area.

### Implications

This research paper is an addition to the discussion of the discourses of scholarly material of southwestern Arabia that has not been discussed enough in the Arabic literature, which is a discourse of the Westerners. Using a single theoretical framework of a synthesis of the Saidian critique, Bhabhan ambivalence, and Prattian contact zones, it offers a thinking unit of how the people and societies of this region were perceived by the West in the most critical moment of their social and political history.

By connecting the Orientalist discourse with the geographical and political realities of southwestern Arabia, the study contributes to the critical comprehension of the issue. The significance of the first half of the twentieth century as a turning point in history that led to the creation of modern states, the ascent and fall of the European imperial presence, and the appearance of the new monarchies in the Gulf predisposes it as an appropriate object of the type of discourse analysis that is historically based and is conducted here.

### Recommendations

The study recommends intensifying critical studies of Orientalist discourses addressing the Arabian Peninsula particularly its southwest to deconstruct the cultural and political patterns upon which they were built. It is advisable to employ multiple approaches in studying travelers’ writings semiotic analysis, cultural criticism, discourse analysis to understand the ideological contents hidden behind the geographical or ethnographic narrative.

It is also recommended that books not available in Arabic, such as the writings of Zogmayr, Cornwallis, and Leibniz, be translated and published academically as part of a series devoted to the history of the Arabian Peninsula in the writings of Western travelers.

Possible future research could usefully include: (1) a comparison of the tribal social organization in northern and southern Arabian travel writing, on whether the respective political situation in each derives systematically different representational methodologies; (2) a discourse-analysis of the particular function of military/intelligence personnel in shaping British policy toward the Arabian Peninsula, based on archive materials as well as published accounts; (3) How such writings were read, reviewed, and circulated within institutions in Britain and Europe during the period under study, considered in terms of enabling contextual conditions, and (4) a comparison of native Arabic narratives of encounters with British travelers at the turn of the century, that answers to the sort of contrapuntal reading which attends to dominant and subaltern perspectives, as Said himself insisted must be the antidote to Orientalist critique.

## Data Availability

The data supporting the conclusions of this article will be made available by the authors. Requests to access these datasets should be directed to dr.e0057s@gmail.com.
